# Stem Cell-Based Therapies in Chagasic Cardiomyopathy

**DOI:** 10.1155/2015/436314

**Published:** 2015-06-15

**Authors:** Antonio Carlos Campos de Carvalho, Adriana Bastos Carvalho

**Affiliations:** Instituto de Biofísica Carlos Chagas Filho, Universidade Federal do Rio de Janeiro, Rua Carlos Chagas Filho 373, 21949-900 Rio de Janeiro, RJ, Brazil

## Abstract

Chagas disease is caused by *Trypanosoma cruzi* and can lead to a dilated cardiomyopathy decades after the prime infection by the parasite. As with other dilated cardiomyopathies, conventional pharmacologic therapies are not always effective and as heart failure progresses patients need heart transplantation. Therefore alternative therapies are highly desirable and cell-based therapies have been investigated in preclinical and clinical studies. In this paper we review the main findings of such studies and discuss future directions for stem cell-based therapies in chronic chagasic cardiomyopathy.

## 1. Introduction

Chagas disease is caused by the hemoflagellate parasite* Trypanosoma cruzi* [1]. The disease is endemic in Latin America and currently there are 8 million people infected, most in endemic areas [2] but the disease has reached North America and Europe, where 300 and 200 thousand people have been infected through blood transfusions [3]. Chagas disease has an acute and a chronic phase. The acute phase is characterized by intense parasitemia, which often goes undetected since patients are asymptomatic or oligosymptomatic. The chronic phase, when parasitemia disappears, is characterized by the indeterminate period, in which patients are asymptomatic for years or decades, or by gastrointestinal and/or cardiac alterations. Cardiac manifestations are more prevalent, reaching 30% of all infected individuals, and are composed of arrhythmias and heart failure, the main causes of death in chagasic patients [4]. The pathogenesis of chronic chagasic cardiomyopathy (CCC) is a matter of debate to these days, but parasite persistence, autoimmune mechanisms, autonomic dysfunction, and microcirculatory alterations have been proposed as the main physiopathologic causes of the disease [5]. Whatever the pathogenic mechanism may be, hearts of chronic chagasic patients and animals show intense inflammatory infiltrate, leading to myocytolysis and myonecrosis and to fibrosis [6], which in turn result in arrhythmias and dilated cardiomyopathy. When heart failure ensues pharmacologic therapy may not prevent deterioration of cardiac function requiring patients to be submitted to heart transplantation. Due to the shortage of donors and complications related to immune suppression, especially in patients affected by a parasitic disease, alternative therapies have been actively pursued for CCC. In that scenario cell-based therapies emerged as a possibility at the turn of the century and were explored in animal models.

## 2. Preclinical Studies

**Figure 1:**
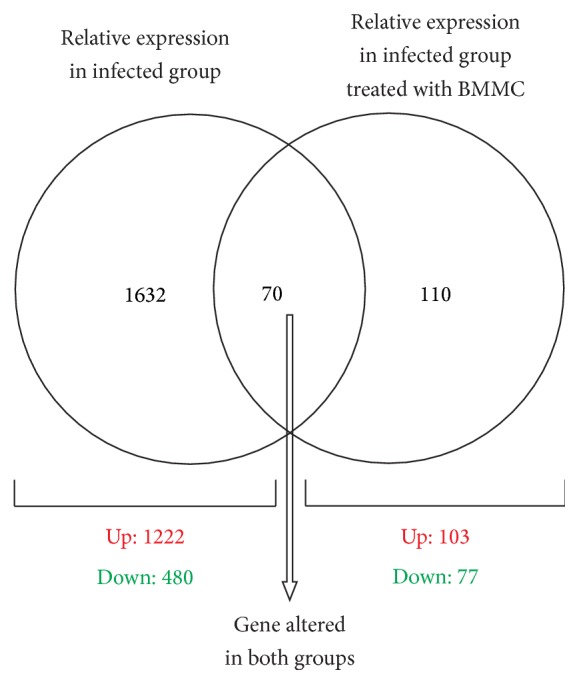
Venn diagram showing the number of cardiac genes altered in each experimental group. Relative expression was calculated using noninfected mice. Analysis was performed eight months after intraperitoneal injection of 1000 trypomastigotes in both groups and 2 months after treatment in the bone marrow mononuclear cell- (BMMC-) treated group. The seventy genes represented at the intersection of the two spheres were altered in both experimental groups. In the left and right, the total number of genes that had their expression solely affected in the infected and BMMC-treated animals is listed. In total, 1702 genes out of 9390 represented in the array had their expression altered by infection—1222 upregulated and 480 downregulated. When infected animals were treated with the cells, only 180 genes had their expression altered when compared to control—103 upregulated and 77 downregulated.

We have used the mouse model for our studies of cell-based therapies in Chagas disease. Different mouse and* T. cruzi *strains were used [7, 8]. In all cases after 4–12-month infection, inflammation and fibrosis were detected in hearts of all infected animals. In addition, Goldenberg et al. [8] showed right ventricular dilatation in the infected animals. Cell therapy in the infected animals was performed using the mononuclear fraction of the bone marrow from syngeneic donors. This fraction was obtained by Ficoll gradient centrifugation and contains a very low percentage of hematopoietic and mesenchymal stem cells (less than 2%). Cells were injected intravenously and the first question addressed was whether they reached the hearts of the infected animals. We used bone marrow from enhanced green fluorescent protein (EGFP) transgenic mice and showed by histopathology that the cells migrated to the infected mouse hearts [7]. In order to emulate an autologous cell therapy in translating our studies to chagasic patients we also used bone marrow cells from chronically infected mice. Using noninfected or chagasic donor bone marrow, mononuclear cell injection resulted in significant reduction in inflammation and fibrosis, as determined by histopathology [7]. Cell dosing revealed that at least 10^5^ cells were necessary for this effect and that a single cell injection resulted in a prolonged beneficial effect (6 months). When morphologic measurements were performed using magnetic resonance imaging (MRI), we observed that mononuclear cell therapy prevented and reversed the right ventricular dilatation characteristic of chronically infected mice [8]. Furthermore, we showed that mononuclear cell therapy reversed cardiac gene expression alterations induced by chronic infection [9]. This effect, as shown in Figure 1, resulted in normalization of more than 85% of the cardiac genes whose expression was altered by the infection.

The rat has also been used as a model for cell-based therapies in chagasic cardiomyopathy. Guarita-Souza et al. [10] injected cocultured skeletal myoblasts and mesenchymal bone marrow derived cells directly into the left ventricle of chronically chagasic rats and reported improvement in left ventricular ejection fraction and decreased end-systolic and end-diastolic volumes by echocardiography.

Although the results with mononuclear cell therapy in the mouse models were highly significant, we wanted to explore additional cell types for therapy of CCC. Among these, the use of mesenchymal stem cells (MSC) seemed to us particularly interesting, given the fact that MSC were reported to have immune modulatory effects [11] and CCC involves inflammation and autoimmune mechanisms targeting the heart. Furthermore, MSC were reported to differentiate into cardiomyocytes under specific culture conditions [12]. We labeled bone marrow derived MSC with fluorescent nanoparticles and tracked them using an* in vivo *imaging system. We showed that a significantly higher number of intravenous injected cells homed to the hearts of infected animals when compared to controls, although most cells were found in the liver, lungs, and spleen [13]. Furthermore, the injected MSC were shown to reduce right ventricular dilatation by micro-PET and MRI analysis [13, 14], as previously found using mononuclear cells. MSC therapy also brought levels of connexin 43 and cytokines (IL-10 and IFN-*γ*), which had been altered by infection, closer to control levels. Interestingly, MSC injection significantly increased SDF-1 levels in the heart. All these effects were attributable to paracrine effects of the injected cells, since we could find no evidence of the labeled cells differentiating into cardiomyocytes. In addition, experiments in which we injected MSC transduced with luciferase 2 (Luc2) and tracked the cells* in vivo* by bioluminescence, after luciferin injection, showed that luminescence could not be detected in the animals after one week (unpublished results).

## 3. Clinical Trials

The exciting results obtained in mouse models led to a clinical trial to investigate the safety of mononuclear cell injection in patients with CCC. The group led by Vilas-Boas [15] enrolled 28 patients with congestive heart failure due to Chagas disease in an open label, single center, uncontrolled phase 1 trial. Inclusion criteria admitted patients from both genders, with ages between 20 and 70 years, left ventricle ejection fraction (LVEF) less than 40%, NYHA class III or IV, being in optimal pharmacologic therapy, and being clinically stable for at least one month prior to enrollment. Patients were subjected to bone marrow aspiration in the morning of the procedure, when 50 mL of marrow was aspirated and processed by Ficoll gradient centrifugation to obtain the mononuclear fraction. This BMMC fraction was resuspended in 20 mL of saline solution and slowly injected in the coronary arteries. Twenty-five days later patients received daily injections of granulocyte colony stimulating factor (g-CSF) at 5 *μ*g/kg for 5 days. The injection of g-CSF was included in the clinical protocol based on unpublished results that showed additive effects of this cytokine when injected after BMMC in a mouse model, as well as in the results reported by Macambira et al. [16] showing that three cycles of 5-day consecutive applications of g-CSF decreased inflammation and fibrosis and increased oxygen consumption in chronically infected mice.

Patients were followed for two months after cell injection and no adverse events that could be directly related to the procedure could be detected. Arrhythmias were a major safety concern, due to their high frequency in chagasic patients, but no change was detected in the arrhythmogenic profile 24 hours after cell injection. Three patients died during the second month of follow-up, but deaths could not be causally associated to cell transplantation according to the authors. Overall, this trial showed the procedure to be feasible and safe, while suggesting indications of functional and clinical improvement for the patients (small but significant increase in LVEF and 6-minute walking test; decrease in NYHA score and Minnesota Living with Heart Failure Questionnaire).

In another smaller trial we investigated retention of the BMMC delivered by intracoronary route in the hearts of 6 CCC patients. Cells were labeled with technetium 99 m before injection and tracked by scintigraphy [17]. Cell retention was measured at 1, 3, and 24 hours after injection, showing a retention of 5.4%, 4.3%, and 2.3%, respectively, of the total radioactivity in the heart.

Based on these results we decided to perform a phase II/III trial to test the efficacy of BMMC in patients with CCC. The efficacy trial was named the MiHeart study and it was a prospective, randomized, multicenter, double-blinded, and placebo-controlled trial. We enrolled 234 patients, of both genders, with ages 18–75 years, NYHA classes II–IV, and LVEF less than 35%. Inclusion and exclusion criteria can be found in the original publication [18]. It is of note that all patients were subject to bone marrow aspiration and ischemic lesions were considered as exclusion criteria, demanding that all patients be subject to coronary angiography before randomization. While patients were in CAT lab, they were randomized (if no coronary lumen obstruction greater than 50% was found) and BMMC in saline containing 5% autologous serum or just saline with 5% autologous serum was delivered by intracoronary injection. A minimum of 100 million cells was injected in patients randomized to the BMMC group. Because of follow-up losses and protocol violations we analyzed data from 183 patients that completed the stipulated one-year follow-up. The primary endpoint was the difference in LVEF from baseline to 6 and 12 months after treatment between cell and placebo-treated groups. Analysis of the data collected was performed by intention to treat analysis and powered to detect a 5-point difference in LVEF between groups. The results showed that both groups, BMMC and placebo, improved LVEF at 6 and 12 months follow-up, but there was no significant difference between the two groups. This same pattern was observed in secondary endpoints, such as six-minute walking distance, brain natriuretic peptide (BNP), NYHA class, and Minnesota Questionnaire. We concluded that the procedure was safe but that injection of autologous BMMC in patients with CCC did not bring additional benefits when compared to standard pharmacologic therapy.

## 4. Lost in Translation?

After the results of the clinical trial, the obvious question is why did the striking results in mouse models not translate to humans?

Mice have been the most used animal models for studies of CCC. They reproduce faithfully the immunologic and histologic alterations found in the human disease, such as immune cell activation, production of cytokines and chemokines, and tissue inflammation and fibrosis [19–21]. Morphologic and functional alterations have been explored less frequently, due to limited availability of imaging systems with adequate resolution for the small dimensions of the mouse heart. Nonetheless, the first evaluations of morphologic and functional alterations of infected mouse hearts were performed using low frequency probes in clinical grade ultrasound equipment and suggested a decrease of left ventricular fractional shortening in chronically infected animals [22]. With the availability of imaging equipment with greater resolutions—high frequency probes for ultrasound and magnetic resonance imaging of higher field intensity—it became clear that* T. cruzi* infection in mice resulted in right ventricular dilatation without significant changes in left ventricular morphology or function [8, 23]. These morphologic and functional alterations in infected mice seem to be independent of the mouse or* T. cruzi* strain used. It is thus clear that the mouse model does not faithfully reproduce the human disease, and this may explain why we had such significant results in the preclinical studies and failed to observe any benefit in the human study.

Another important difference between the mouse models and the human trials is that patients are treated pharmacologically and therapy is optimized before inclusion. In the mouse models we are observing the effects of the cells in hearts that have not been exposed to drugs. In patients, cell therapy would have to add to the pharmacologic therapy. But of course there are other reasons that may explain our failure to translate the mouse model to humans. Cell number, for instance, has been proposed as determinant for a positive effect. In the MiHeart study, we did not inject the same number of cells in all patients; this number varied by as much as 8 times, and we could not find correlation between cell number and cardiac improvement. We therefore think unlikely that this might explain the failure in humans. Furthermore, there have been reports in the literature that more cells may not necessarily imply better results [24]. Disease state may also influence outcome: we chose to test BMMC therapy in advanced stage CCC patients and we feel that for an experimental therapy this was the most appropriate and ethical decision at the time. However, BMMC therapy could be effective in patients in which the disease is less severe. Route of delivery is another important variable determining outcome of cell-based trials. We chose the intracoronary route, a compromise between intravenous and intramyocardial routes, since exclusion criteria demanded a coronary angiography and cell injection was performed at that time, thus avoiding two invasive procedures. Positive results have been reported in human trials using all three routes [25–27], suggesting that this may not be an essential determinant of outcome. Last but not least, cell type may explain our failure, even though BMMC were effective in the mouse model. The mononuclear fraction of the bone marrow contains very few stem cells. At any rate, bone marrow derived stem cells are not capable of differentiating into cardiomyocytes and, therefore, if the effects of cell therapies are attributable to paracrine secretion of factors, the BMMC fraction may be as competent as a pure stem cell population to secrete these factors. As mentioned before, we think that MSC may constitute an interesting cell population to test in CCC. In the mouse model these cells proved to be effective, but given that BMMC were also effective and did not translate to humans, we see no reason for starting a new human trial based on the mouse results.

## 5. Future Directions

Based on the need to offer CCC patients alternative therapies to heart transplantation we started a preclinical study using chagasic dogs. Chronically infected dogs develop a cardiomyopathy similar to the human disease. After 9 months of infection, LVEF is significantly decreased, cardiac inflammation and fibrosis are prominent, and cytokines and antibodies present in human disease are found in the dog model [28, 29]. Since BMMC failed in the human trial, we are testing bone marrow derived mesenchymal stem cells in the dog studies. We are testing both autologous and allogeneic MSC in dogs chronically infected with* T. cruzi,* which have a significant decrease in LEVF. Once this initial study is concluded and if there are positive results with either of or both cell types, we will start a second study where we will treat the infected dogs pharmacologically with the same drugs commonly used to treat heart failure in patients. After optimizing pharmacologic therapy, based on analysis of LVEF, we will then treat a group with MSC (either autologous or allogeneic) while the other group will receive only the pharmacologic therapy. If we observe significant additional improvement in the cell-treated group we will then proceed to propose a new clinical trial for CCC patients. We expect to conclude the initial studies with the chronically infected dogs by the end of this year and the studies with the drug treated animals by the end of 2016.

Meanwhile we are still using the mouse model to study the effects of other cell types and other delivery routes in CCC. We are testing adipose tissue-derived MSC and pluripotent cells predifferentiated into cardiomyocytes in the chronically infected mouse model using both the intravenous and an echo guided intramyocardial injection route. Furthermore, using a double transgenic mouse model, as described by Loffredo and coworkers [30], we are investigating the generation of new cardiomyocytes during the course of* T. cruzi* infection. We hope that these studies will provide the basis for successful translational studies in the future and that we will find the proper cell type, cell number, disease stage, and injection route to promote a significant benefit for CCC patients.

## References

[1] Chagas C. (1909). Nova tripanozomiaze humana: estudos sobre a morfolojia e o ciclo evolutivo do *Schizotrypanum cruzi* n. gen., n. sp., ajente etiolojico de nova entidade morbida do homem. *Memórias do Instituto Oswaldo Cruz*.

[2] Nunes M. C. P., Dones W., Morillo C. A., Encina J. J., Ribeiro A. L. (2013). Chagas disease: an overview of clinical and epidemiological aspects. *Journal of the American College of Cardiology*.

[3] Coura J. R., Vĩas P. A. (2010). Chagas disease: a new worldwide challenge. *Nature*.

[4] Rassi A., Marin-Neto J. A. (2010). Chagas disease. *The Lancet*.

[5] Tanowitz H. B., Machado F. S., Jelicks L. A. (2009). Perspectives on *Trypanosoma cruzi*-induced heart disease (Chagas disease). *Progress in Cardiovascular Diseases*.

[6] Marin-Neto J. A., Cunha-Neto E., Maciel B. C., Simões M. V. (2007). Pathogenesis of chronic Chagas heart disease. *Circulation*.

[7] Soares M. B. P., Lima R. S., Rocha L. L. (2004). Transplanted bone marrow cells repair heart tissue and reduce myocarditis in chronic chagasic mice. *American Journal of Pathology*.

[8] Goldenberg R. C. S., Jelicks L. A., Fortes F. S. A. (2008). Bone marrow cell therapy ameliorates and reverses chagasic cardiomyopathy in a mouse model. *Journal of Infectious Diseases*.

[9] Soares M. B. P., Lima R. S., Souza B. S. F. (2011). Reversion of gene expression alterations in hearts of mice with chronic chagasic cardiomyopathy after transplantation of bone marrow cells. *Cell Cycle*.

[10] Guarita-Souza L. C., Carvalho K. A. T., Woitowicz V. (2006). Simultaneous autologous transplantation of cocultured mesenchymal stem cells and skeletal myoblasts improves ventricular function in a murine model of Chagas disease. *Circulation*.

[11] Tolar J., le Blanc K., Keating A., Blazar B. R. (2010). Concise review: hitting the right spot with mesenchymal stromal cells. *Stem Cells*.

[12] Makino S., Fukuda K., Miyoshi S. (1999). Cardiomyocytes can be generated from marrow stromal cells in vitro. *Journal of Clinical Investigation*.

[13] Jasmin, Jelicks L. A., Koba W. (2012). Mesenchymal bone marrow cell therapy in a mouse model of Chagas disease. Where do the cells go?. *PLoS Neglected Tropical Diseases*.

[14] Jasmin J., Jelicks L. A., Tanowitz H. B. (2014). Molecular imaging, biodistribution and efficacy of mesenchymal bone marrow cell therapy in a mouse model of Chagas disease. *Microbes and Infection*.

[15] Vilas-Boas F., Feitosa G. S., Soares M. B. P. (2006). Early results of bone marrow cell transplantation to the myocardium of patients with heart failure due to chagas disease. *Arquivos Brasileiros de Cardiologia*.

[16] Macambira S. G., Vasconcelos J. F., Costa C. R. S. (2009). Granulocyte colony-stimulating factor treatment in chronic Chagas disease: preservation and improvement of cardiac structure and function. *The FASEB Journal*.

[17] da Fonseca L. M. B., Xavier S. S., de Castro P. H. R. (2011). Biodistribution of bone marrow mononuclear cells in chronic chagasic cardiomyopathy after intracoronary injection. *International Journal of Cardiology*.

[18] dos Santos R. R., Rassi S., Feitosa G. (2012). Cell therapy in chagas cardiomyopathy (Chagas arm of the multicenter randomized trial of cell therapy in cardiopathies study): a multicenter randomized trial. *Circulation*.

[19] Guedes P. M. D. M., Gutierrez F. R. S., Maia F. L. (2010). IL-17 produced during *Trypanosoma cruzi* infection plays a central role in regulating parasite-induced myocarditis. *PLoS Neglected Tropical Diseases*.

[20] Kierszenbaum F. (2005). Where do we stand on the autoimmunity hypothesis of Chagas disease?. *Trends in Parasitology*.

[21] Dutra W. O., Rocha M. O. C., Teixeira M. M. (2005). The clinical immunology of human Chagas disease. *Trends in Parasitology*.

[22] Chandra M., Shirani J., Shtutin V. (2002). Cardioprotective effects of verapamil on myocardial structure and function in a murine model of chronic *Trypanosoma cruzi* infection (Brazil Strain): an echocardiographic study. *International Journal for Parasitology*.

[23] de Souza A. P., Tang B., Tanowitz H. B., Araújo-Jorge T. C., Jelicks E. L. A. (2005). Magnetic resonance imaging in experimental Chagas disease: a brief review of the utility of the method for monitoring right ventricular chamber dilatation. *Parasitology Research*.

[24] Losordo D. W., Henry T. D., Davidson C. (2011). Intramyocardial, autologous CD34^+^ cell therapy for refractory angina. *Circulation Research*.

[25] Schächinger V., Erbs S., Elsässer A. (2006). Intracoronary bone marrow-derived progenitor cells in acute myocardial infarction. *The New England Journal of Medicine*.

[26] Hare J. M., Fishman J. E., Gerstenblith G. (2012). Comparison of allogeneic vs autologous bone marrow–derived mesenchymal stem cells delivered by transendocardial injection in patients with ischemic cardiomyopathy: the POSEIDON randomized trial. *The Journal of the American Medical Association*.

[27] Perin E. C., Dohmann H. F. R., Borojevic R. (2004). Improved exercise capacity and ischemia 6 and 12 months after transendocardial injection of autologous bone marrow mononuclear cells for ischemic cardiomyopathy. *Circulation*.

[28] Guedes P. M. M., Veloso V. M., Afonso L. C. C. (2009). Development of chronic cardiomyopathy in canine Chagas disease correlates with high IFN-*γ*, TNF-*α*, and low IL-10 production during the acute infection phase. *Veterinary Immunology and Immunopathology*.

[29] Daliry A., Caldas I. S., Diniz L. D. F. (2014). Anti-adrenergic and muscarinic receptor autoantibodies in a canine model of Chagas disease and their modulation by benznidazole. *International Journal of Cardiology*.

[30] Loffredo F. S., Steinhauser M. L., Gannon J., Lee R. T. (2011). Bone marrow-derived cell therapy stimulates endogenous cardiomyocyte progenitors and promotes cardiac repair. *Cell Stem Cell*.

